# Obesity prevalence estimates in a Canadian regional population of preschool children using variant growth references

**DOI:** 10.1186/1471-2431-11-21

**Published:** 2011-02-28

**Authors:** Laurie K Twells, Leigh A Newhook

**Affiliations:** 1 School of Pharmacy, Memorial University, 300 Prince Philip Drive, St. John's, NL, A1B 3V6, Canada; 2 Faculty of Medicine, Memorial University, 300 Prince Philip Drive, St. John's, NL, A1B 3V6, Canada

## Abstract

**Background:**

Childhood obesity is a public health problem in Canada. Accurate measurement of a health problem is crucial in defining its burden. The objective of this study is to compare the prevalence estimates of overweight and obesity in preschool children using three growth references.

**Methods:**

Weights and heights were measured on 1026 preschool children born in Newfoundland and Labrador (NL), Canada, and body mass index calculated. The prevalence of overweight and obesity was determined and statistical comparisons conducted among the three growth references; the Centres for Disease Control (CDC), the International Obesity Task Force (IOTF) and the World Health Organization (WHO).

**Results:**

CDC and IOTF produced similar estimates of the prevalence of overweight, 19.1% versus 18.2% while the WHO reported a higher prevalence 26.7% (p < .001). The CDC classified twice as many children as obese compared to the IOTF 16.6% versus 8.3% (p < .001) and a third more than the WHO 16.6% versus 11.3% (p < .01). There was variable level of agreement between methods.

**Conclusions:**

The CDC reported a much higher prevalence of obesity compared to the other references. The prevalence of childhood obesity is dependent on the growth reference used.

## Background

Globally, obesity is a significant public health problem [1,2] and a number of studies report an increasing prevalence of overweight and obese children in Canada [3-6]. The health risks associated with excess body weight are well documented [7,8]. The age and sex specific body mass index (kg/m^2^) or BMI is the most common method for assessing weight status and health risk in children [9]. There are three sets of growth references commonly used to assess a child's weight status and health risk; BMI cut-points published by the US Centre for Disease Control and Prevention (CDC), the International Obesity Task Force (IOTF) and those published by the World Health Organization (WHO) [10-12]. Inconsistent prevalence estimates of childhood overweight and obesity based on variant growth references pose a challenge in defining the burden of childhood obesity at a population level. Recommendations are inconsistent on which method to use [13,14].

The purpose of this paper is to compare prevalence estimates of overweight and obesity among a regional preschool population living in the province of Newfoundland and Labrador, Canada using the CDC, IOTF and WHO BMI cut-points. A secondary objective is to assess the level of agreement between the growth references.

## Methods

The Memorial University Human Investigations Committee and the Health and Community Services Boards ethics committees approved this study.

### Study Design & Population

This is cross-sectional analysis of 1026 children (mean age 4.5 years) living in the province of Newfoundland and Labrador who participated in pre-Kindergarten Health Fairs prior to starting school in 2005. The Fairs were open to all children and provided immunizations and tests for vision, hearing and developmental problems. The population is described elsewhere [15]. Two trained research staff collected the information required for the current study.

### Data Collection and Study Variables

Research assistants trained by a Pediatrician took direct anthropometric measures. Children were asked to take off their shoes for the height measure and to take off any over clothing for the weight measure. Direct measures of weight were collected using a Tanita digital weighing scale (kg) rounded to one decimal place calibrated to the hospital digital scale. An Invicta stadiometer (cm) was used to measure the height rounded to one decimal place of the children. Two measures were taken and the average recorded. Sex and age in years and months were collected and rounded to the nearest half year.

### Defining overweight and obesity

For each child BMI (kg/m^2^) was calculated and classified according to the cut-points published by the CDC, IOTF and the WHO.

#### The US Centre for Disease Control

The US CDC publishes BMI age and sex-specific growth references derived from five nationally representative surveys of American children conducted between 1963 and 1994 [10]. Using software downloaded with permission from the CDC, children were classified as overweight (BMI >85^th ^) and obese (BMI ≥95^th^) [16].

#### The International Obesity Task Force

In 2000, the IOTF published BMI cut-points for defining overweight and obesity in children between 2 and 18 years [11]. These references are based on children living in six countries (i.e., United States, Brazil, Great Britain, Hong Kong, Netherlands, Singapore) and can be extrapolated to the widely accepted definitions for adult overweight and obesity; a BMI ≥ 25 and a BMI ≥ 30 respectively, shown to be predictive of adverse health outcomes in adults. Using these cut-points, a preschool child is considered overweight with a BMI ≥ 91^st ^and obese with a BMI ≥ 99^th ^percentile, respectively.

#### The World Health Organization

In 2006, the WHO released new growth references for assessing and monitoring the growth in children. These were generated from data collected from the WHO Multicentre Growth Reference Study in six countries (i.e., Brazil, Ghana, India, Oman, Norway, United States). Children under study were raised in optimal conditions that included living in a nonsmoking environment. The children were exclusively or predominantly breastfed for more than four months, fed complementary foods by six months, had continuation of breastfeeding until at least 12 months, were immunized and had access to and received required healthcare. Using the BMI-for-age z-scores, children were classified as overweight with a BMI between one and two standard deviations (SD) above the mean and obese with a BMI more than two SDs above the mean. Overweight in this population is classified as a BMI >84^th ^percentile while a BMI >97.7^th ^percentile classifies a child as obese [12].

### Statistical Analysis

Continuous variables were normally distributed and reported using means and standard deviations. Categorical data were reported as whole numbers and percentages. Statistical comparisons were conducted using student t-tests for continuous data and chi-squared analysis for categorical data. Cohen's kappa statistic was calculated to determine the level of agreement between the growth references. A kappa greater than .80 signifies very good agreement, between .60-.80 a good level of agreement and that less than .50 little to moderate agreement [17]. A p-value <.05 was statistically significant. All data was analyzed using the Statistical Package for the Social Sciences (SPSS 15.0).

## Results

**Figure 1:**
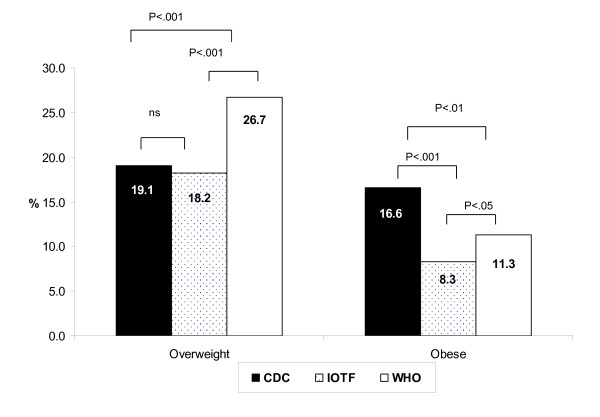
**Prevalence of overweight and obesity using CDC, IOTF and WHO growth references**. CDC: The Centres for Disease Control and Prevention, IOTF: The International Obesity Task Force, WHO: The World Health Organization. p < .05 significant, ns: not significant

The anthropometric measures and characteristics of the children are presented in Table 1. There were no significant differences in these variables. Figure 1 illustrates that for the overweight group, there were significant differences between CDC and WHO (19.1% and 26.7%) and between IOTF and WHO (18.2% and 26.7%). When children were classified as obese, there were significant differences across the three references (CDC 16.6%, IOTF 8.3%, WHO 11.3%).

**Table 1:** Characteristics of study sample (n = 1026)

	Boys	Girls	Total
Sample size	537	489	1026
Age in years mean ± SD	4.5(0.5)	4.5(0.5)	4.49 (0.5)
Height, cm, mean ± SD	110.4(5.2)	110.5(5.2)	110.3 (5.2)
Weight, kg, mean ± SD	20.5(3.5)	20.2(3.9)	20.4 (3.7)
BMI (kg/m^2^), mean ± SD	16.7(1.9)	16.6(2.3)	16.7 (2.1)

BMI: Body Mass Index, WHO: The World Health Organization, IOTF: The International Obesity Task Force, CDC: The Centre for Disease Control and Prevention, SD: Standard Deviation

**Figure 2:**
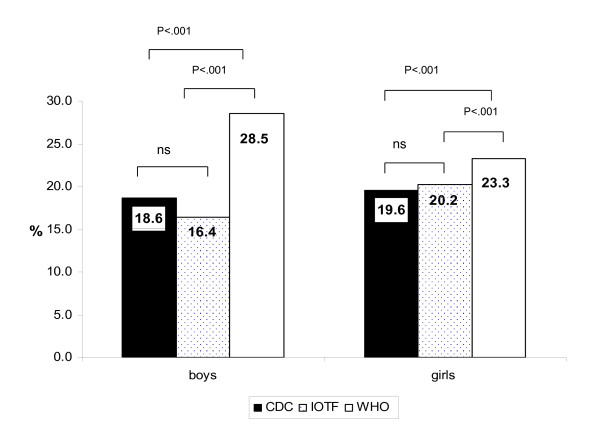
**Prevalence of overweight in boys and girls using CDC, IOTF and WHO growth references**. CDC: The Centres for Disease Control and Prevention, IOTF: The International Obesity Task Force, WHO: The World Health Organization. p < .05 significant, ns: not significant

**Figure 3:**
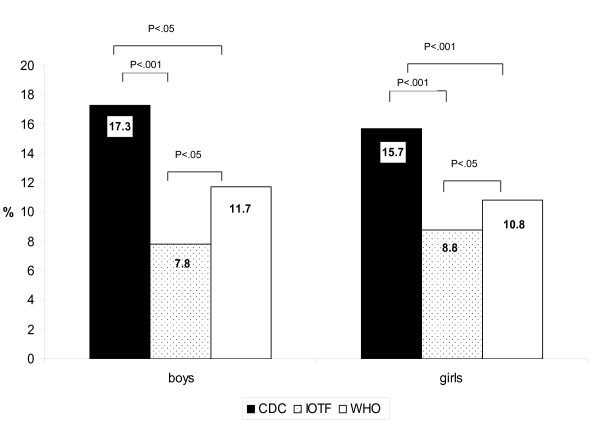
**Prevalence of obesity in boys and girls using CDC, IOTF and WHO growth references**. CDC: The Centres for Disease Control and Prevention, IOTF: The International Obesity Task Force, WHO: The World Health Organization. p < . 05 significant, ns: not significant

In Figure 2, for boys, there were significant differences in overweight between IOTF and WHO (16.4% vs. 28.5%) and between CDC and WHO (18.6% vs. 28.5%). A similar relationship was found for girls. In Figure 3, there were significant differences in the obese category across all three standards for both boys (CDC 17.3%, IOTF 7.8%, WHO 11.7%) and girls (CDC 15.7%, IOTF 8.8%, WHO 10.8%). To determine the level of agreement between growth references, we calculated the kappa statistic (Table 2).

**Table 2:** Comparison of agreement for categorizing weight categories between 1) CDC and WHO 2) IOTF and WHO and 3) CDC and IOTF

1.		WHO			
**CDC**		**Normal**	**Overweight**	**Obese**	**Total**
	Normal	634	26	0	660
	Overweight	8	188	0	196
	Obese	1	53	116	170
	Total	643	267	116	1026

% agreement: 91%, kappa statistic: .84, p < .001					

**2.**		**WHO**			

**IOTF**		**Normal**	**Overweight**	**Obese**	**Total**
	Normal	643	111	0	754
	Overweight	0	156	31	187
	Obese	0	0	85	85
	Total	643	267	116	1026

% agreement: 86%, kappa statistic: .71, p < .001					

**3.**		**IOTF**			

**CDC**		**Normal**	**Overweight**	**Obese**	**Total**
	Normal	659	1	0	660
	Overweight	91	105	0	196
	Obese	4	81	85	170
	Total	754	187	85	1026

% agreement: 83%, kappa statistic: .64, p < .001					

WHO: The World Health Organization, IOTF: The International Obesity Task Force, CDC: The Centre for Disease Control and Prevention, p < .05 significant

## Discussion

There is a high prevalence of childhood overweight or obesity in this Canadian preschool population, irregardless of growth reference used. Approximately one in three preschool children was overweight or obese. Similar statistics are being reported in other developed counties [18-27]. The short and long term physical health risks for children associated with excess weight include hypertension, hyperinsulinemia, glucose intolerance, type II diabetes, dyslipidemia, increased risk of early cardiac disease and psychosocial difficulties. Childhood obesity is a significant public health concern and accurate measurement and classification is crucial in determining the burden of the health problem. In the current study, the CDC reported a significantly higher prevalence of childhood obesity compared to the other growth references. The CDC reported more than double the prevalence of obesity compared to the IOTF and approximately 30% more obese than the WHO. Whether this is an accurate reflection or an overestimation of the prevalence of obesity in this population, it is difficult to tell. Conflicting recommendations are provided by various organizations that include the Canadian Pediatric Society [28] and the Clinical Practice Guidelines on the management and prevention of obesity in adults and children [13]. Although there was an overall good level of agreement between the different growth references there was some noticeable inconsistencies in classification which may lead to challenges in assessing the weight status of individual children in a clinical environment.

Several studies are published comparing the prevalence estimates of overweight and obesity in children and youth using various growth references, and the findings are inconsistent [18-27]. One study recently published on a sample of Canadian children and youth compared the estimates of excess weight using the same three reference cut-points as in the current study and similar findings were reported. The prevalence estimate for the childhood obesity estimate was the same based on the WHO and CDC growth references (13%) but lower based on the IOTF cut-points (8%) [18]. Another study on children between four and six years of age living in the province of Alberta, compared prevalence estimates using CDC and IOTF cut-points. The authors reported that an estimated 13.8% and 11.4% of children were overweight and obese according to the CDC cut-offs, while an estimated 11.5% and 6.8% of children were classified as overweight and obese using the IOTF cut-offs. Similar to the current study, the CDC growth references classified almost twice as many children as obese compared to the IOTF method. The level of agreement between the two methods was .69 (p < .01), significantly lower then that in the current study [22].

In an Italian study on 258 preschool children three to six year of age, the prevalence of overweight and obesity were compared using the CDC and IOTF methods as well as local Italian growth references published by Luciano [23]. All three sets of growth references gave similar estimates of overweight in boys (CDC 16.10%, IOTF 12.90%, Luciano 14.5%) and girls (CDC 15.70%, IOTF 15.7%, Luciano 10%). These findings were not dissimilar to the current study's findings on overweight in boys (CDC 18.6%, IOTF 16.4%) and overweight in girls (CDC 19.6%, IOTF 20.2%). However in the Italian study, the use of the CDC reference led to a prevalence estimate of obesity in boys that was ten times that of the other references (CDC 10.5%, IOTF 0.8%, Luciano 0.8%). In girls the CDC estimate was also significantly higher compared to the other two references (CDC 11.90%, IOTF 6.7%, Luciano 3%), although not as large a difference as in the boys. Based on this study it appears that the reliability of the growth reference used may be affected by the underlying prevalence of childhood obesity in the population, as the Italian rates of childhood obesity tend to be much lower than those found in North America. Ethnic diversity will also have an impact. This provides further challenges when making international comparisons [23]. In contrast to the previous studies, a study recently conducted on 604 Spanish children 6 to 10 years of age, researchers reported that when using the WHO criteria, the combined prevalence of overweight and obesity was 39%, significantly higher than both the CDC estimate of 20% and the IOTF estimate of 17%. In this study, the CDC and IOTF demonstrated a high level of agreement (kappa >.80), however the level of agreement between the CDC and the IOTF and the WHO was poor (kappa < .40). In this study, the authors concluded that it was the WHO criteria that overestimated the prevalence of childhood overweight and obesity, not the CDC as seen in the current study [19].

The significant difference in prevalence estimates of childhood obesity produced by the three growth references make it difficult; to assess the extent of the problem and its associated health burden, to conduct research, to make population comparisons and to inform policy. The CDC and to a more limited extent the WHO references appear to allow for an earlier identification of a larger number of children affected by excess weight compared to the IOTF. If the CDC classifies children accurately than using this reference may prompt health professionals to provide primary prevention to a pediatric population earlier to help reduce the risk of associated health conditions [9]. Although the association between increasing BMI and increased health risk has been substantiated in adults [29] it is important to raise a concern that none of these growth references have been convincingly linked to the future development of adverse health outcomes in children.

An important strength of our study is that we directly measured children's heights and weights and did not rely on self reported survey data which may underestimate the prevalence of overweight and obesity. Our study limitations included a non random sample from one region in Canada. However, the prevalence estimates of overweight and obesity in the current study were very similar to a larger provincial study [5], providing confidence that our study findings are representative of this provincial population. The current research adds to the debate about the relative reliability and validity of the variant growth references used to monitor a child's growth and has implications for future research. It raises several research questions that still need to be answered. Have we determined the ideal definition of overweight and obesity in children so that prevention efforts can be initiated at the earliest opportunity? Are some of these references better suited to particular populations depending on the mix of ethnicity and race? Can we validate these growth references against a gold standard? Should references based on optimal growth be considered the gold standard? Are any of these standards associated with adverse health outcomes *in children*? It is clear that more research including longitudinal studies is needed in order to answer many of these questions.

## Conclusions

Childhood obesity is a public health problem and is associated with increased morbidity and mortality. Obesity tracks through the life cycle [2,30] suggesting that early identification and primary prevention is key to reversing and preventing the upward trend into adult obesity and its potential future burden of illness [31]. A consensus is urgently needed on the most valid and reliable growth reference to use to measure and monitor a child's growth for both clinical and research purposes.

## Abbreviations

(NL): Newfoundland and Labrador; (BMI): body mass index; (CDC): Centre of Disease Control; (IOTF): International Obesity Task Force; (WHO); World Health Organization.

## Competing interests

The authors declare that they have no competing interests.

## Authors' contributions

LT was the principal investigator on the project and responsible for the intellectual conception and design of the study including the data analysis and interpretation of the data. LT was also the lead investigator on both the initial funding application the manuscript preparation and provided final approval for submission. LN contributed to the conception and design of the study including preparation of the funding application. LN also helped draft and revise the final manuscript. Both authors have approved the final version of the manuscript for publication.

## Pre-publication history

The pre-publication history for this paper can be accessed here:

http://www.biomedcentral.com/1471-2431/11/21/prepub
